# Intrinsic brainstem circuits comprised of Chx10-expressing neurons contribute to reticulospinal output in mice

**DOI:** 10.1152/jn.00322.2021

**Published:** 2021-10-20

**Authors:** Jeremy W. Chopek, Ying Zhang, Robert M. Brownstone

**Affiliations:** ^1^Department of Medical Neuroscience, Faculty of Medicine, Dalhousie University, Halifax, Nova Scotia, Canada; ^2^Department of Neuromuscular Diseases, UCL Queen Square Institute of Neurology, University College London, London, United Kingdom; ^3^Department of Physiology and Pathophysiology, Rady Faculty of Health Sciences, University of Manitoba, Winnipeg, Manitoba, Canada

**Keywords:** interneurons, locomotion, microcircuits, reticular formation, spinal cord

## Abstract

Glutamatergic reticulospinal neurons in the gigantocellular reticular nucleus (GRN) of the medullary reticular formation can function as command neurons, transmitting motor commands to spinal cord circuits to instruct movement. Recent advances in our understanding of this neuron-dense region have been facilitated by the discovery of expression of the transcriptional regulator, Chx10, in excitatory reticulospinal neurons. Here, we address the capacity of local circuitry in the GRN to contribute to reticulospinal output. We define two subpopulations of Chx10-expressing neurons in this region, based on distinct electrophysiological properties and soma size (small and large), and show that these populations correspond to local interneurons and reticulospinal neurons, respectively. Using focal release of caged glutamate combined with patch clamp recordings, we demonstrated that Chx10 neurons form microcircuits in which the Chx10 local interneurons project to and facilitate the firing of Chx10 reticulospinal neurons. We discuss the implications of these microcircuits in terms of movement selection.

**NEW & NOTEWORTHY** Reticulospinal neurons in the medullary reticular formation integrate inputs from higher regions to effectively instruct spinal motor circuits. Using photoactivation of neurons in brainstem slices, we studied connectivity of reticular formation neurons that express the transcriptional regulator, Chx10. We show that a subpopulation of these neurons functions as local interneurons that affect descending commands. The results shed light on the internal organization and microcircuit formation of reticular formation neurons.

## Graphical abstract

**Figure:**
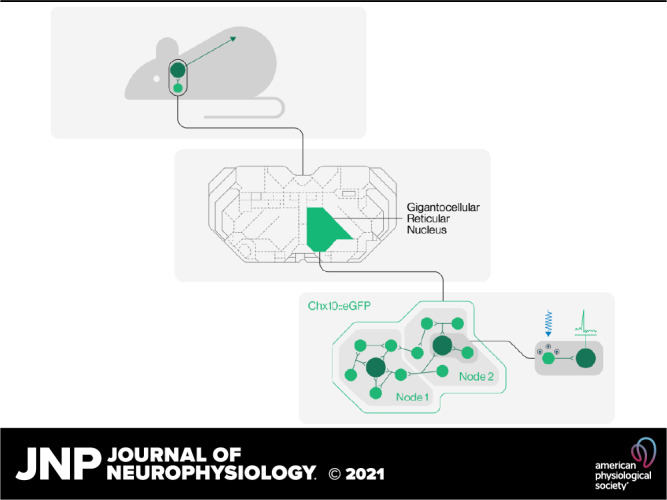

## INTRODUCTION

Movement is complicated. When we reach and grasp our coffee cup, we instruct our spinal cord to activate particular muscle groups in a precise temporal sequence. At the same time, we instruct it to not supinate our forearm. This combination of instructions to move and to not move is thus required for behavioral output. Although the cortex and basal ganglia are involved in making these selections (1, 2), it is the brainstem reticular formation that must ultimately decode these supramedullary inputs to form precise commands that descend, via reticulospinal pathways, to spinal motor circuits (3).

It is well established that the gigantocellular reticular nucleus (GRN) contains reticulospinal neurons (RSNs) that mediate posture and locomotion in the cat (4–6). Neurons in supramedullary locomotor regions such as the mesencephalic (MLR) and cerebellar locomotor regions project to the GRN, where they are integrated to initiate locomotion via reticulospinal pathways that in turn activate spinal locomotor circuits (7, 8). Electrical (9) or chemical (10) activation of the GRN is sufficient for inducing locomotor activity, and inactivation (8) of this region eliminates MLR-evoked locomotion. But glutamatergic RSNs are involved in other movements as well: for example, these neurons also receive input from postural centers to ensure appropriate extensor tone during motor activities (for review, see Ref. 11).

The GRN comprises various types of neurons, including inhibitory as well as excitatory RSNs (12–14), and is not seemingly structurally organized in a way that would facilitate the study of different neuronal types. Mouse genetic techniques have therefore been used to identify and activate (or inactivate) neurons in this region to study their roles. In recent years, neurons in the GRN that express the transcriptional regulator Chx10 have been studied (15, 16). As in the spinal cord (17, 18), these neurons are exclusively glutamatergic (15, 19). Promoters for either the vesicular glutamate transporter 2 (vGluT2) or Chx10 have been used to drive cre recombinase expression for opto- or chemogenetic experiments. Using these techniques, it has become clear that excitatory glutamatergic GRN neurons can modulate locomotor activity (20, 21), that bilateral activation of Chx10 reticular formation neurons can stop locomotion (19), and that unilateral stimulation of Chx10 or glutamatergic reticular formation neurons can lead to turning (22) or neck movements (23), respectively, or both (24). But glutamatergic neurons in this region are also involved in sleep atonia (25), suggesting that results from photoactivation experiments could be skewed by the subpopulation(s) of neurons activated. It is likely that, similar to the heterogeneity of spinal Chx10 interneurons (e.g., see Refs. 26 and 27), Chx10 reticular formation neurons are also not homogeneous (24, 28). Furthermore, opto- or chemogenetic studies would not capture the intrinsic organization of the GRN, and whether local circuits regulate the output—that is, whether this region is simply an integrator of inputs, or whether it has the circuitry to refine descending output.

In a step toward understanding this black box region of the brain and how it might process inputs from higher brain centers to produce descending commands for spinal cord circuits to process, we asked whether Chx10 neurons in the GRN form local microcircuits. We identified two clear subsets of these neurons, only one of which (comprised of neurons with large somata) is reticulospinal. Then, using focal release of caged glutamate, we demonstrated the internal architecture whereby smaller Chx10 neurons project to and facilitate the firing of the larger Chx10 neurons. We propose that these microcircuits tune descending commands and thus facilitate movement.

## MATERIALS AND METHODS

### Animals

All animal procedures were done at Dalhousie University, approved by the University Committee on Laboratory Animals as well as the University College London (UCL) Animal Welfare Ethical Review Body, and conform to the standards of the Canadian Council for Animal Care and UK Home Office Project License 70/9098. Both male and female BAC Chx10::eGFP [STOCK Tg(Vsx2-EGFP)FJ99Gsat/Mmucd] mice (available from the Mutant Mouse Resource and Research Centers, MMRRC No. 011391-UCD) maintained on a Swiss Webster background were used in all experiments. This line has been used previously to study spinal V2a neurons with high specificity (29) and reticular formation neurons, where green fluorescent protein (GFP) expression was shown to have high specificity for Chx10-expressing neurons (15).

### Electrophysiology

#### Slice preparations.

Whole cell patch clamp recordings were made from visualized Chx10 neurons from the rostral GRN of the medulla of P9–P14 Chx10e:GFP mice (15). This is an age when locomotor function is becoming mature (30). Mice were anesthetized by intraperitoneal injection of ketamine (60 mg/kg) and xylazine (12 mg/kg). After loss of their righting reflex, mice were cooled on ice and decapitated. The brainstem was immediately removed and secured to an agar block with a small amount of adhesive and transferred to ice-cold oxygenated solution (3.5 mM KCL, 35 mM NaHCO_3_, 1.2 mM KH_2_PO_4_, 1.3 mM MgSO_4_, 1.2 mM CaCl_2_, 10 mM glucose, 212.5 mM sucrose, 2 mM MgCl_2_, pH7.4) for sectioning. Two, 300–350 μm sections of the medulla containing the rostral GRN were sectioned on a vibratome (Leica VT1200S, Leica) per mouse and transferred to a warm (30°C) oxygenated (95% oxygen, 5% carbon) artificial cerebrospinal fluid (aCSF) solution (111 mM NaCl, 3.08 mM KCl, 11 mM Glucose, 25 mM NaHCO_3_, 1.25 mM MgSO_4_, 2.52 mM CaCl_2_, 1.18 mM KH_2_PO_4_, pH 7.4) to recover for a minimum of 30 min.

#### Whole cell patch-clamp recordings and stimulation.

Slices were transferred to a recording chamber mounted on a Zeiss AxioExaminer microscope and perfused with oxygenated room-temperature aCSF. Cells were visualized using a ×20 wide aperture (1.2 nA) water-immersion objective lens, a CCD camera (CoolSNAP EZ CCD Camera, Photometrics, AZ) and Slidebook 6.0 software (Intelligent Imaging Innovations, CO, RRID:SCR_014300).

Whole cell patch-clamp recordings were acquired in current-clamp (IC) mode using a Multiclamp 700B amplifier (Molecular Devices, San Jose, CA, RRID:SCR_014300). Recordings were loss pass filtered at 10 kHz (IC) and acquired at 25 kHz with CED Power1401 AD board and Signal software (CED, Cambridge, UK, http://ced.co.uk/us/products/sigovin). Recording pipettes were filled with a solution containing in mM: K-gluconate, 128; NaCl, 4; CaCl_2_, 0.0001; HEPES, 10 mM, glucose, 1 mM; Mg-ATP, 5; and GTP-Li, 0.3, pH 7.2, lucifer yellow dilithium salt (0.4 mg/mL, Thermo Fisher Scientific, Cat. No. A-5750, RRID:AB_2314410), and neurobiotin (1 mg/mL, Vector Laboratories, Cat. No. SP-1120-20, RRID: AB_2536191), and had resistances of 4–6 MΩ. Lucifer yellow allowed for the immediate visualization of the soma and dendrites of the patched cell. This allowed us to avoid photolysis over processes of the recorded cell while stimulating nearby presynaptic cell bodies. Neurobiotin allowed for post hoc confirmation of the identity of the recorded cell and that axons or dendrites of the postsynaptic cell were not in the regions of presynaptic cell bodies, such that they would be stimulated. This was facilitated by comparing images of the region of interest (ROIs) stimulated (see *Immunohistochemistry and Imaging*) to the confocal image of the biotin-filled postsynaptic cell (*n* = 10).

In the initial set of experiments, basic and rhythmic firing properties of visually identified “large” and “small” Chx10 neurons in the rostral GRN were subjected to various depolarizing and hyperpolarzing current pulses. All properties were collected while holding the cell at −60 mV. Input resistance was collected as the average response of the cell to repetitive (minimum 20 sweeps), small hyperpolarizing pulses (−10 pA, 100 ms). Rheobase, defined as the minimum current required to elicit an action potential 50% of time was collected from incremental 1 pA depolarizing current steps. Voltage threshold defined as the membrane potential at which depolarization increased at ≥10 V/s (31) was determined from the first spiking response during rheobase. Frequency-current (*F*-*I*) plots were obtained by applying 1-s depolarizing current pulses with incremental increases of 10–20 pA. The steady-state firing frequency was determined by counting the number of spikes during the 1-s pulse. The initial firing frequency was collected by taking the average interspike interval (1/average interspike interval) of the first three spikes for each current step. Spike frequency adaptation (SFA) was calculated as the percent of reduction in firing rate from the initial to the steady-state firing frequency. Sag potential and postinhibitory rebound firing was collected during 1-s hyperpolarizing current pulses with increases of −10 to −20 pA. Sag potential was collected as the difference in the membrane potential at peak minus steady-state change during the hyperpolarizing pulse. All sag calculations were conducted at a steady-state hyperpolarization of −100 mV.

### Holographic Photolysis of Caged Glutamate

Following the initial set of experiments to characterize the electrophysiological properties of large and small Chx10 neurons, we sought to determine the connectivity patterns of Chx10 neurons using a photolysis protocol as we have previously described (32). These experiments only proceeded when membrane potential was stable (i.e., did not fluctuate more than 5 mV during a 5-min period). 4-Methoxy-7-nitroindolinyl-caged-L-glutamate (MNI-glutamate) (2.5 mM, Tocris, ON, Canada, Cat. No. 1490) was perfused in warm (30°C) aCSF at a rate of 2 mL/min. Holographic photolysis of MNI-glutamate was performed using a 405-nm laser directed through a Phasor spatial light modulator (SLM) system (Intelligent Imaging Innovations, CO), stimulating regions of interest (ROI) as controlled by Slidebook software. We have used this method previously, validating that photolysis beyond 7 µm from the neuron, photolysis did not elicit a response—that is, there were no nonspecific effects resulting from glutamate diffusion (32). Before photostimulating ROIs, direct photolysis over the patched Chx10 neuron was performed to determine the intensity and duration of the pulse which elicited a single action potential. Ideal intensity and pulse duration for small Chx10 neurons was 3.5 mW and 800–1,000 µs and for large Chx10 neurons was 7 mW and 1,500 µs, calculated by the percentage of the maximum laser output that was measured with a power meter in the absence of tissue. These parameters did not evoke responses in the absence of perfused caged glutamate. If longer durations were necessary, the slice was not used as it was deemed to be unhealthy. ROIs were selected as identified neuronal cell bodies under fluorescence, with the ROI blanketing the soma. Once ROIs in a single focal plane were selected, sequential photostimulation of the ROIs was carried out while recording from the postsynaptic neuron. Postsynaptic cells were held between −60 mV and −50 mV for the duration of the experiment (which could exceed 1 h). Photomicrographs for each ROI were taken to map the location of each cell relative to the postsynaptic cell recorded. In stable preparations, once a connection was established, a second electrode was used to record from the connected presynaptic cell to determine if the connection was bidirectional. As the connected cells were often in close proximity (100–200 μm), the gigaseal of the patch-clamp was often lost on one of the two cells, but continued with loose patch recordings as confirmed by persisting responses from direct photolysis over the cell body.

### Cholera Toxin Subunit B Injections

The fluorescent retrograde tracer cholera toxin subunit B (CTB) was used to label reticulospinal neurons. Briefly, using aseptic surgery with 3%–4% isoflurane, an incision over the cervical or lumbar spine was made, the lamina over C7 or L2 was removed, and a small incision in the dura was made to expose the spinal cord. Mice were placed in a stereotaxic frame to stabilize the vertebral column and, using a micromanipulator, a glass pipette containing 1 µL of CTB was advanced to the surface of the spinal cord, following which the tip was slowly lowered 500 µm into the C7 or L2 spinal cord. CTB was injected over a 10-min period and the pipette remained in place for 5 min after injection, after which, the electrode was removed and a similar injection with a new glass electrode was made on the contralateral side of the spinal cord. The mice were then sutured and monitored while they recovered. No deficits or motor impairments were seen as mice recovered.

### Immunohistochemistry and Imaging

Upon completion of the electrophysiological recordings, slices were incubated in 4% paraformaldehyde for 1 h at room temperature followed by three, 15-min washes in 0.1% PBS-T. Slices were incubated at 4°C overnight in goat anti-GFP primary antibody (1:2,500, Abcam Cat. No. AB6673, RRID: AB_305643), with well-established specificity, followed by a 4-h incubation period in donkey anti-goat Alexa Fluor 488 (1:500, Abcam Cat. No. AB150105, RRID: AB_2732856) and Alexa Fluor 647-conjugated streptavidin (1:500, Thermo Fisher Scientific, Cat. No. S-21374. RRID: AB_2336066). Images were obtained using a Zeiss LSM 510 upright confocal microscope. Images were compared with photos obtained during the electrophysiology recordings in Slidebook to confirm that the ROIs did not overlap with visually identifiable dendrites or axons.

For CTB-labeled tissue, the mice were anesthetized 14 days after injection and transcardially perfused with phosphate buffer solutions followed by 4% paraformaldehyde in 0.1 M phosphate solution (PBS). Tissue was harvested, postfixed in 4% paraformaldehyde overnight, and subsequently cryoprotected in 30% sucrose. Tissue was sectioned (30 µm) using a cryostat and mounted on glass slides for immunohistochemistry. Sections were incubated with primary goat anti-GFP (1:500, Abcam, Cat. No. AB5449, RRID: AB_304896) antibodies diluted in PBS containing 0.1% Triton X-100 (PBS-T) for 24 h at 4°C. Sections were then washed three times for 10 min each in 0.1% PBS-T followed by incubation with appropriate secondary antibodies conjugated to Alexa 488, Alexa 555, and dapi-405 (1:500 Molecular Probes) for 3 h. Sections were then washed three times for 10 min each in PBS, mounted in Vectashield (Vector Laboratories) and cover-slipped. Epifluorescent images were acquired with a Zeiss Axioplan inverted microscope. For quantification of Chx10 soma sizes, the 15–20 sections through the rostral GRN were examined, and cells were only counted when their nuclei (visualized with DAPI staining) were clearly present. Cell areas (µm^2^) were calculated using surface area function in ImageJ. Confocal images (20 µm z-stacks) were subsequently obtained and illustrated with representative single optical sections in Fig. 2.

### Experimental Design and Statistical Analysis

Data are presented as means ± SD. As is common for discovery experiments, no statistical method was used to predetermine sample size, and no randomization or blinding procedures were used. Statistical analyses were done using Sigma-Plot (v14.0, Systat software, CA). Shapiro–Wilk tests were used to confirm normal distributions. As such, unpaired Student’s *t* tests were used for all comparisons except for frequency of observed sag potentials, in which Fisher Exact test was used. Statistical significance was set at *P* < 0.05.

## RESULTS

### Chx10 Neurons Have Distinct Electrophysiological Properties Correlated to Their Size

As intrinsic neuronal electrophysiological properties play a critical role in producing circuit activity (33), we first sought to characterize these properties of medial medullary reticular formation (medRF) Chx10 neurons, focusing on those in the rostral GRN. We performed whole cell patch-clamp recording from Chx10 neurons in P9–P14 mice to determine if there are subpopulations of Chx10 neurons with distinct electrophysiological properties. Once stable, all neurons were held at −60 mV to have a uniform baseline when measuring their responses to injected current.

**Figure 1.**
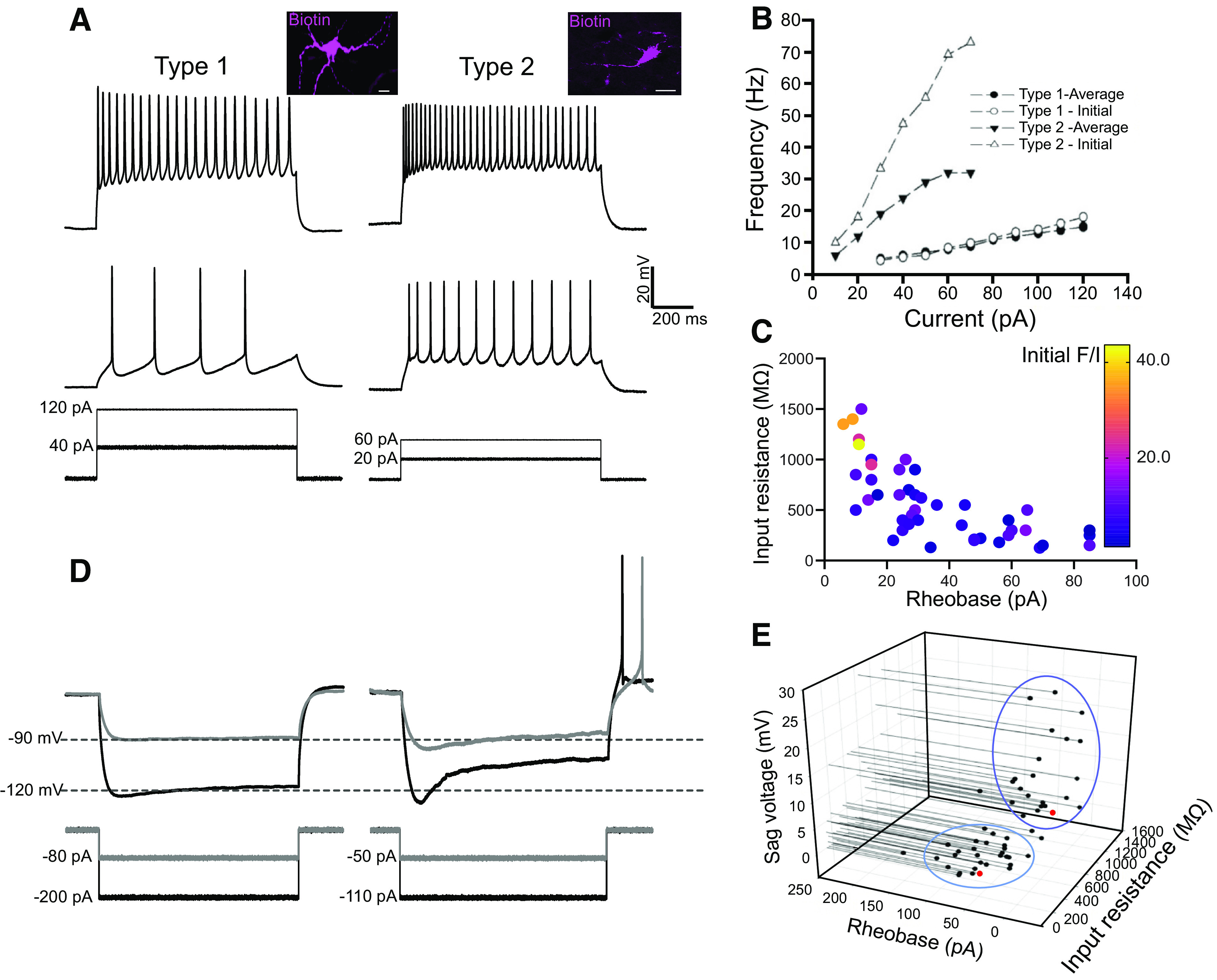
Chx10 neurons have two distinct types of electrophysiological properties. *A*: in response to incremental depolarizing current steps, *Type 1* Chx10 neurons demonstrate minimal gain in their firing rate with little spike frequency adaptation, whereas *Type 2* Chx10 neurons demonstrate a high initial firing rate with spike frequency adaptation (SFA). *Insets* of biotin filled Chx10 neurons imaged post hoc with confocal microscope. Scale bars = 20 µm. *B*: frequency-current relationship plots of the initial and average firing rates for *Type 1* and *Type 2* Chx10 neurons. *C*: relationship of initial frequency-current (*F*-*I*) slope with input resistance and rheobase. Color reflects the *F*-*I* gain (Hz/10 pA; see sidebar). *D*: in response to incremental hyperpolarizing current steps, *Type 1* Chx10 neurons demonstrate minimal sag and no postinhibitory rebound (PIR), whereas *Type 2* Chx10 neurons demonstrate substantial sag and PIR following termination of the hyperpolarizing pulse. *E*: sag voltage, rheobase, and input resistance recorded from each Chx10 neuron. Blue circles showing separation of *Type 1* and *Type 2* Chx10 neurons based on their plotted electrophysiological properties. The neurons indicated by the two red dots are shown in Fig. 5. Data from 48 neurons collected from 33 slices of 21 mice of both sexes.

We first characterized the spike patterns of Chx10 neurons in response to sustained suprathreshold current steps. Of the 60 neurons we recorded, 48 demonstrated sustained firing in response to current injection, whereas the remaining 12 fired only a single spike with abnormal morphology, suggesting poor health and therefore not analyzed further. Of the 48 neurons with sustained firing, two distinct patterns of firing emerged. The first pattern (*n* = 26, from 13 mice and 18 slices) consisted of trains of action potential with minimal SFA and lower initial gain in the *F*/*I* relationship (Fig. 1, *A* and *B*). The second pattern (*n* = 22, from 8 mice and 15 slices) consisted of high initial firing rate followed with pronounced spike frequency adaptation. The average gain for the initial firing (first 3 spikes) was 19 ± 18 Hz/10 pA for the first pattern of firing Chx10 neurons and 36 ± 28 Hz/10 pA (*P* = 0.02) for the second type of firing Chx10 neurons (Fig. 1*B*). There was no significant difference in the average gain for the steady firing (last 3 spikes), with average gains of 9 ± 9 Hz/10 pA and 12 ± 9 Hz/10 pA respectively (Fig. 1*B*, *P* = 0.08). Furthermore, the second type of firing Chx10 neurons demonstrated significantly more SFA compared with the first type of Chx10 neurons (43 ± 12% vs. 26 ± 20% decrease in firing rates, respectively, *P* = 0.028). Based on these distinct firing patterns, we termed these neurons *Type 1* and *Type 2* Chx10 neurons, and then analyzed their corresponding electrophysiological properties (Fig. 1*C*).

The two types of Chx10 neurons also demonstrated differences in their responses to hyperpolarizing current injection to steady-state voltages of −100 mV. Of the 26 *Type 1* Chx10 neurons, 10 had sag potentials, in response to a hyperpolarizing step to −100 mV, and these were of small amplitude (3.8 ± 5.1 mV). None of these neurons responded with postinhibitory rebound (PIR) upon termination of this step (Fig. 1*D*). Conversely, all *Type 2* Chx10 neurons (*n* = 22) had a large sag potential (21.4 ± 7.5 mV, *P* < 0.001) in response to hyperpolarization (compared with *Type 1*, Fisher Exact *P* < 0.001), and PIR responses consisted of a single or several action potentials at pulse termination (Fig. 1*D*). To summarize, we found that *Type 1* Chx10 neurons demonstrated: *1*) lower gains in their initial firing *F*/*I* relationships, *2*) minimal SFA, *3*) no sag potentials, and *4*) no PIR, whereas *Type 2* Chx10 neurons demonstrated: *1*) higher gains in their initial firing *F*/*I* relationships, *2*) significant SFA, *3*) sag potentials, and *4*) PIR responses. Based on these two distinct types of firing patterns in response to depolarizing and hyperpolarizing current injections, we proceeded to compare passive electrical properties of these two types of Chx10 neurons.

Both types demonstrated similar resting membrane potentials (−46.8 ± 5.4 mV and −46.2 ± 6.6 mV, *Type 1* and *2* respectively, *P* = 0.73) and spike thresholds (−45.2 ± 6.4 mV and −43.4 ± 3.7 mV, *Type 1* and *2* respectively, *P* = 0.23). However, there was a significant difference in input resistance with *Type 1* demonstrating an average input resistance of 380 ± 245 MΩ whereas *Type 2* had an average input resistance of 980 ± 480 MΩ (*P* < 0.001). Correspondingly, *Type 1* Chx10 neurons were less excitable with an average rheobase of 56.0 ± 40.8 pA, compared with *Type 2* Chx10 neurons which had an average rheobase of 21.0 ± 7.5 pA (*P* < 0.001). These three properties—input resistance, rheobase, and sag—together led to a separation between *Type 1* and *Type 2* Chx10 neurons (Fig. 1*E*).

During these recordings, it became visually evident that *Type 1* Chx10 neurons were larger than *Type 2* Chx10 neurons (Fig. 1*A*, *inset*). This observation matched their different rheobases and input resistances. Furthermore, we injected biotin into patched neurons and analyzed their soma sizes and the number of initial dendritic branches (*n* = 5 for each type). It was clear that *Type 1* Chx10 neurons have significantly larger somas (385 ± 48 µm^2^ vs. 148 ± 45 µm^2^, *P* < 0.001) and have more primary dendrites (3.4 ± 0.5 vs. 1.4 ± 0.5, *P* = 0.004) than *Type 2* Chx10 neurons. The difference in soma size fits with a bimodal distribution of Chx10 soma sizes (vide infra). We therefore refer to these two types of GRN neurons as Large Chx10 neurons and Small Chx10 neurons, and next sought to determine their connectivity patterns.

### Large Chx10 Neurons Are Reticulospinal

**Figure 2.**
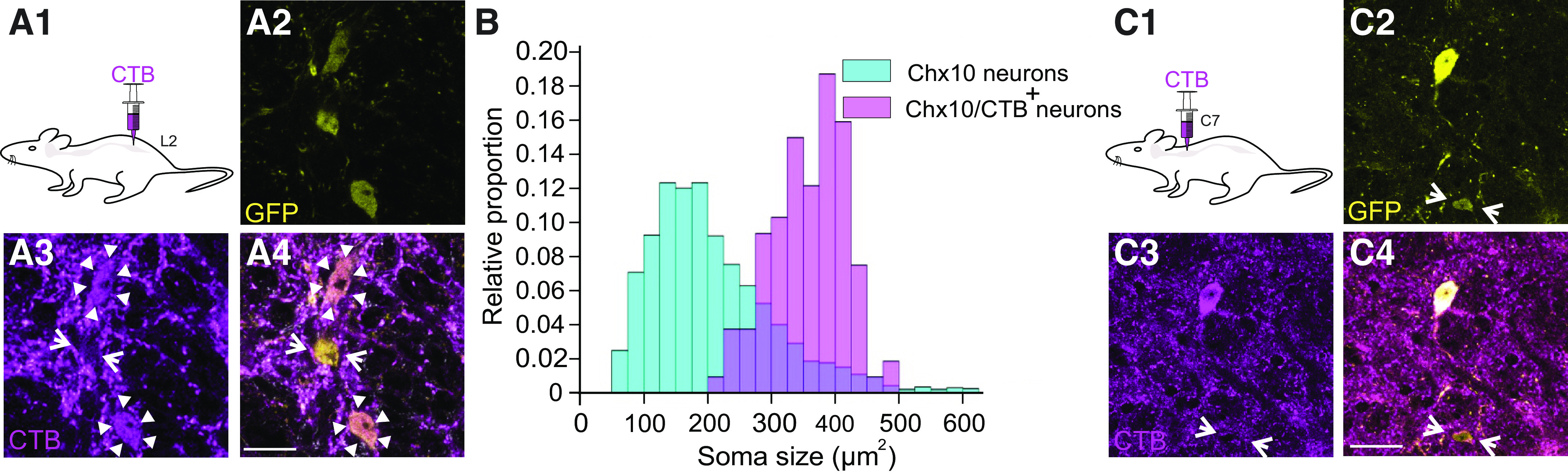
Large Chx10 neurons are reticulospinal neurons. *A1*: schematic of experimental procedure in which cholera toxin subunit B (CTB) was injected bilaterally in the second lumbar segment to label reticulospinal neurons. *A2*: single optical plane image of GFP positive Chx10 neurons in the gigantocellular region of the medulla. *A3*: CTB labeling of terminals and cell bodies in the same optical section as *A2*. *A4*: overlap of GFP and CTB, showing two Chx10 neurons positive for CTB labeling (arrow heads), and one smaller one that is not (arrows). *n* = 3 mice of both sexes. Scale bar = 20 µm. *B*: overlaid cumulative plots demonstrating the distribution of all labeled GFP Chx10 neurons in the gigantocellular reticular nucleus (GRN) (light blue), as well as the distribution of CTB positive Chx10 neurons (pink). The relative proportion indicates the fraction of total observations within each group that falls in each 25 µm bin. Note that although the proportion of large Chx10 cells is lower, it is these large cells that are reticulospinal. *C1*: schematic of experimental procedure in which CTB is injected in the seventh cervical segment to label reticulospinal neurons. *C2*: single optical plane image of GFP positive Chx10 neurons in the gigantocellular region of the medulla. *C3*: CTB labeling of terminals and cell body in the same optical section in *C2*. *C4*: overlap of GFP and CTB, showing one Chx10 neuron positive for CTB and one smaller one that is not (arrow). *n* = 3 mice of both sexes. Scale bar = 30 µm.

As Chx10 neurons have previously been shown to be reticulospinal (15, 19), we sought to determine whether both subpopulations project to the spinal cord. We initially targeted the L2 spinal cord, where we injected CTB bilaterally for retrograde labeling in adult Chx10:eGFP mice (*n* = 3, Fig. 2*A*). After a 2-wk recovery period, we found, on average, 63 (range 35–82) CTB+ GFP^ON^ neurons/mouse in the rostral GRN (Fig. 2*A*). This represented 8%–11% of all Chx10^ON^ neurons in this region of the rostral GRN, similar to the proportion reported in neonatal mice (15). Interestingly, there were many bouton-like structures labeled with CTB, suggesting the presence of a noteworthy spinoreticular projection from the lumbar spinal cord.

As a population, Chx10 soma sizes ranged from 70 to 610 µm^2^ (*n* = 1,925, from 3 mice, mean: 211 ± 105 µm^2^). In contrast, the range of average soma sizes of CTB positive Chx10 neurons was 217–540 µm^2^ (mean: 360 ± 60 µm^2^; *P* < 0 .01), corresponding with larger Chx10 neuronal soma sizes seen in the frequency distribution (Fig. 2*B*). Of the 188 Chx10 neurons (*n* = 3 mice) larger than 300 µm^2^, 156 (83%) were CTB+. These data demonstrate that the Large Chx10 neurons are reticulospinal. We found no evidence that the Small Chx10 neurons project to the lumbar spinal cord.

To investigate whether Small Chx10 neurons project to the rostral spinal cord, we injected CTB in the C7 cervical segments (*n* = 3) and quantified the number of CTB+ Chx10^ON^ neurons in the rostral GRN as aforementioned (Fig. 2*C*). We found a similar distribution of CTB+ Large Chx10 neurons and no CTB+ Small Chx10 neurons. Thus, Large Chx10 neurons are reticulospinal whereas Small Chx10 neurons are not. We will therefore refer to the Large Chx10 neurons as Chx10 RSNs.

### Connectivity Patterns of Chx10 Neurons

To determine whether the two populations are synaptically connected with each other, we used brain stem slice preparations to record from 30 Chx10 neurons (15 of each type), while photo-activating caged glutamate on over 120 others, establishing 60 connections. We targeted Chx10 neurons based on their size to ensure that we recorded a sufficient number of each type. Once entering whole cell mode, we first characterized the neurons based on their electrophysiological properties and found a 96% (29/30) concordance rate between their properties and the predicted type based on size. That is, if we treat our initial data from 48 neurons as a “training” set, then in this “test” set of 30 neurons, we picked their electrophysiological properties correctly in 29. Therefore, we were confident that we could visually differentiate small Chx10 neurons from Chx10 RSNs for photostimulation. As can be appreciated by the distribution of cell sizes (Fig. 2*B*), there were many more small Chx10 neurons than reticulospinal neurons. As demonstrated below, we found that small Chx10 neurons form synapses with other small Chx10 neurons and with Chx10 RSNs. Conversely, we did not find evidence that Chx10 RSNs form synapses with other Chx10 RSNs and connections to small Chx10 neurons were infrequent. In a subset of pairs, we found bidirectional connectivity between small Chx10 neurons and Chx10 RSNs. As small Chx10 neurons projected locally and formed connections with both small Chx10 and Chx10 RSNs, we will refer to these neurons as Chx10 local interneurons. This designation does not negate the possibility that they also have projections to areas beyond the GRN. We describe these findings below.

### Chx10 Local Interneurons Form Synapses with Chx10 Local Interneurons

**Figure 3.**
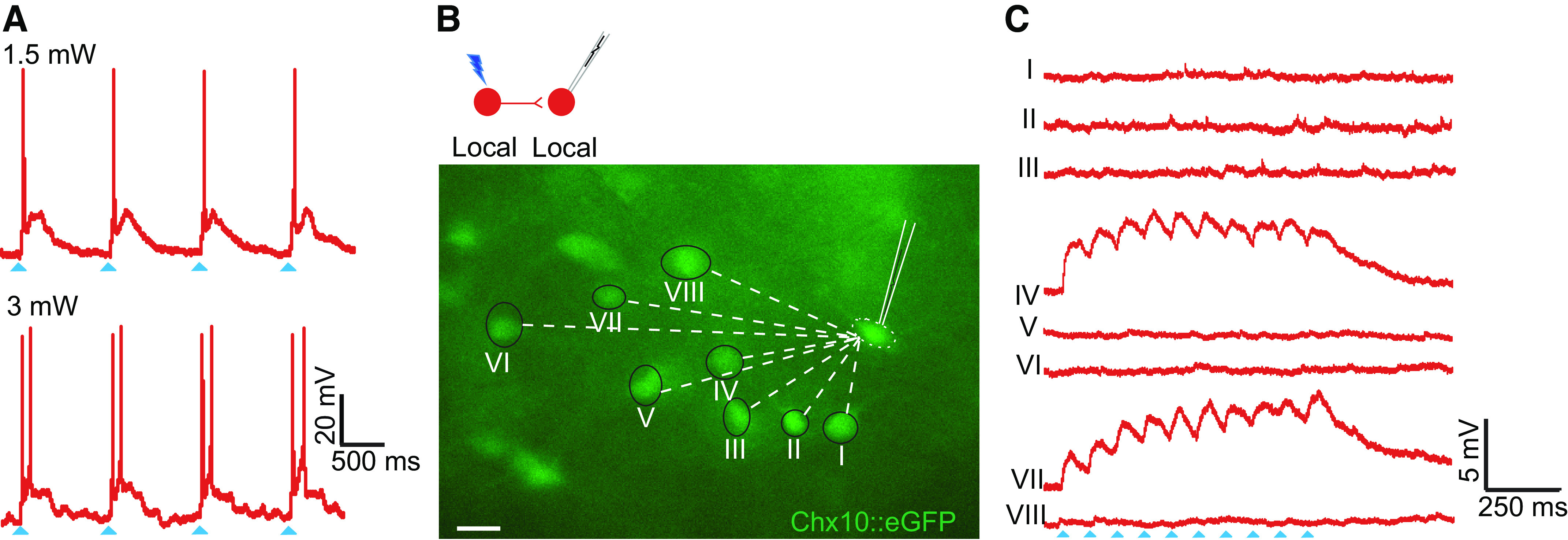
Chx10 local interneurons form synapses with other Chx10 local interneurons. *A*: direct holographic photolysis over a patched Chx10 local interneurons results in single or multiple action potentials, dependent on the laser output. *B*: fluorescence image of Chx10::eGFP positive neurons in the rostral gigantocellular reticular nucleus (GRN). Synaptic connectivity was determined by stimulating visually identified Chx10 interneurons and recording postsynaptic potentials in the patched Chx10 interneuron. Scale bar = 30 µm. *C*: postsynaptic responses from photolysis over Chx10 local interneurons with corresponding numbers in *B*. in this example, stimulation (trains) of neurons IV and VII elicited excitatory postsynaptic potentials (EPSPs), whereas stimulation of the other Chx10 neurons did not. Blue arrowheads indicates laser on. Example from 20 connections from 15 neurons in 12 mice of both sexes.

After ensuring that photolysis produced action potentials in Chx10 local interneurons by directly activating the recorded cell (Fig. 3*A*), we asked whether Chx10 local interneurons connect to each other. In 15 Chx10 local interneurons (average soma size 176 ± 40 µm^2^), 20 connections were found by photostimulation of 70 Chx10 local interneurons (Fig. 3*B*). In all 20 connections, the postsynaptic responses were subthreshold, with differing sizes of excitatory postsynaptic potentials (EPSPs) arising from different presynaptic neurons (Fig. 3*C*). In some cases, temporal summation with repetitive stimulation was evident (Fig. 3*C*). The number of connected presynaptic neurons ranged from 1 to 3 (mean 1.7 ± 0.9), with EPSP amplitudes ranging from 4.0 to 12.5 mV (mean 8 ± 3 mV).

### No Apparent Connectivity between Chx10 Reticulospinal Neurons

**Figure 4.**
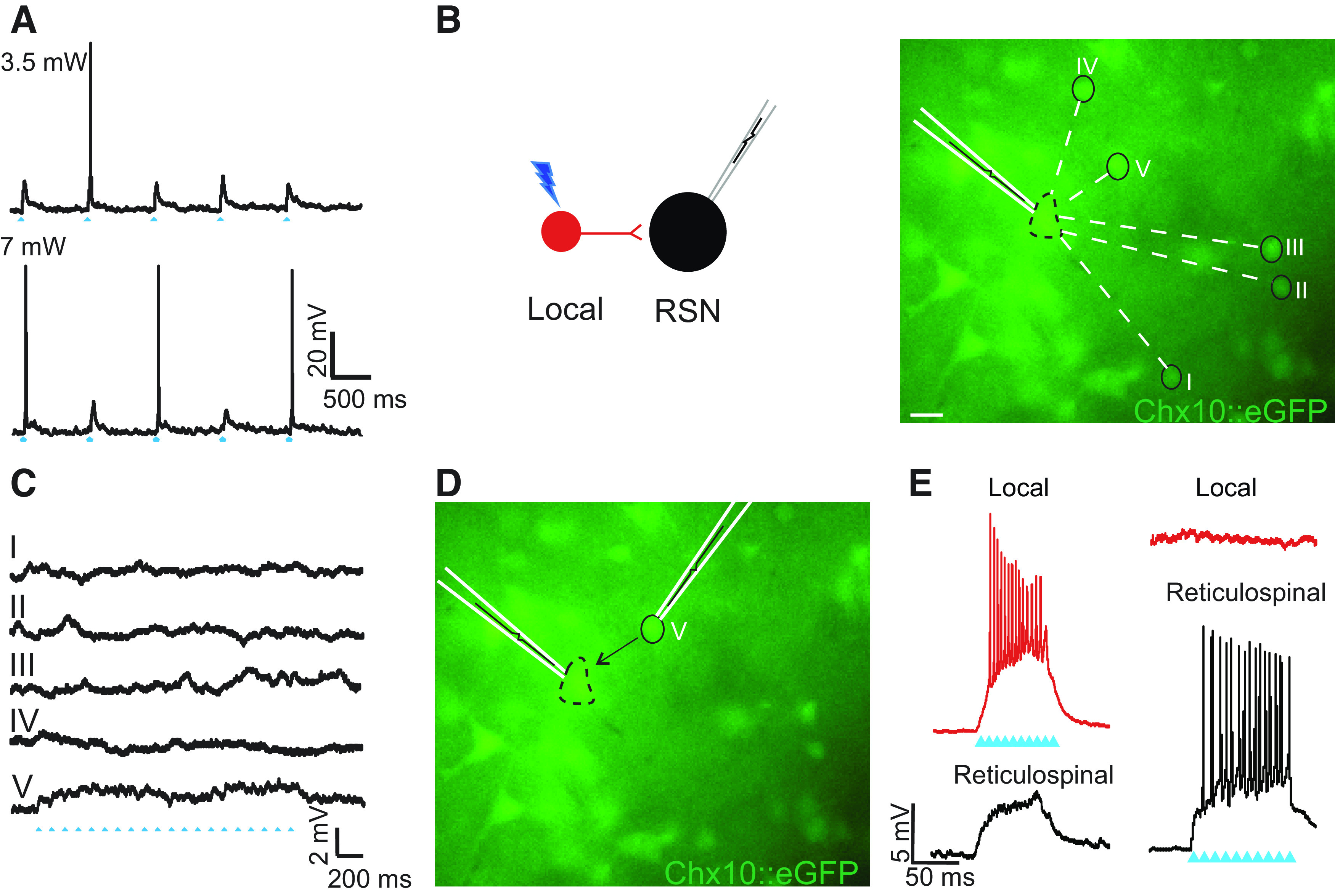
Chx10 local interneurons form synapses with Chx10 reticulospinal neurons. *A*: direct holographic photolysis over a patched Chx10 reticulospinal neuron (RSN) results in excitatory postsynaptic potentials (EPSPs) or single action potentials dependent on laser output. *B*: fluorescence image of Chx10::eGFP positive neurons in the rostral gigantocellular reticular nucleus (GRN). Synaptic connectivity was determined by stimulating visually identified Chx10 local interneurons and recording postsynaptic potentials in the patched Chx10 RSN. Scale bar = 30 µm. *C*: postsynaptic responses from stimulation of numbered Chx10 interneurons. In this example, spot V elicited EPSPs from a train of laser stimuli, whereas stimulation of the remaining Chx10 neurons did not elicit a response. *D*: after establishing a synaptic connection, a second electrode was used to patch and record responses in the Chx10 local interneuron (spot V). Scale bar = 30 µm. *E*: photolysis around the Chx10 local interneuron resulted in multiple action potentials and EPSPs in the Chx10 RSN, whereas photolysis over the Chx10 RSN elicited multiple action potentials but did not elicit a response in the Chx10 local interneuron. Blue arrowheads indicate laser on. Example from 32 connections from 14 pairs of neurons in 11 mice of both sexes.

We next turned to Chx10 RSNs, and asked whether they form synapses with each other. Due to the large size and high rheobase of Chx10 RSNs, higher photostimulation intensities were needed to generate action potentials in response to the majority of the individual stimuli (Fig. 4*A*). During recordings in seven Chx10 RSNs, we photostimulated a total of 40 other Chx10 RSNs but were unable to establish connectivity: there were no responses.

### Chx10 Local Interneurons Form Synapses with Chx10 Reticulospinal Neurons

We next turned to investigating connectivity between the two types of neurons. To study connectivity from Chx10 local interneurons to Chx10 RSNs, we photostimulated 75 Chx10 local interneurons while recording Chx10 RSNs (*n* = 15, soma size 421 ± 84 μm^2^), and found a total of 32 connections in 14 neuron pairs (Fig. 4*B*). In all 32 connections, the postsynaptic responses were subthreshold, resulting in small EPSPs (3.2 ± 1.2 mV, Fig. 4*C*, presynaptic neuron V). The number of connections ranged from 1 to 6 presynaptic neurons, with an average of 2.5 ± 1.3 connections per patched Chx10 RSN. Conversely, in five Chx10 local interneurons, we photostimulated a total of 25 Chx10 RSNs but were unable to establish any connections, suggesting that connectivity between the two populations is unidirectional.

In a subset of experiments that showed connectivity from a Chx10 local interneuron to a Chx10 RSNs (*n* = 7), a second patch electrode was used to successfully record the presynaptic Chx10 local interneuron, although this often resulted in the loss of the initial giga seal on the postsynaptic Chx10 RSN (Fig. 4*D*). In these experiments, action potentials generated in the Chx10 local interneurons by direct photolysis led to EPSPs in the Chx10 RSN as expected (Fig. 4*E*). In 5/7 of these experiments, photostimulation of Chx10 RSN that resulted in the generation of action potentials in the Chx10 reticulospinal neurons did not elicit postsynaptic responses in the Chx10 local interneurons, supporting that these connections are predominantly unidirectional.

**Figure 5.**
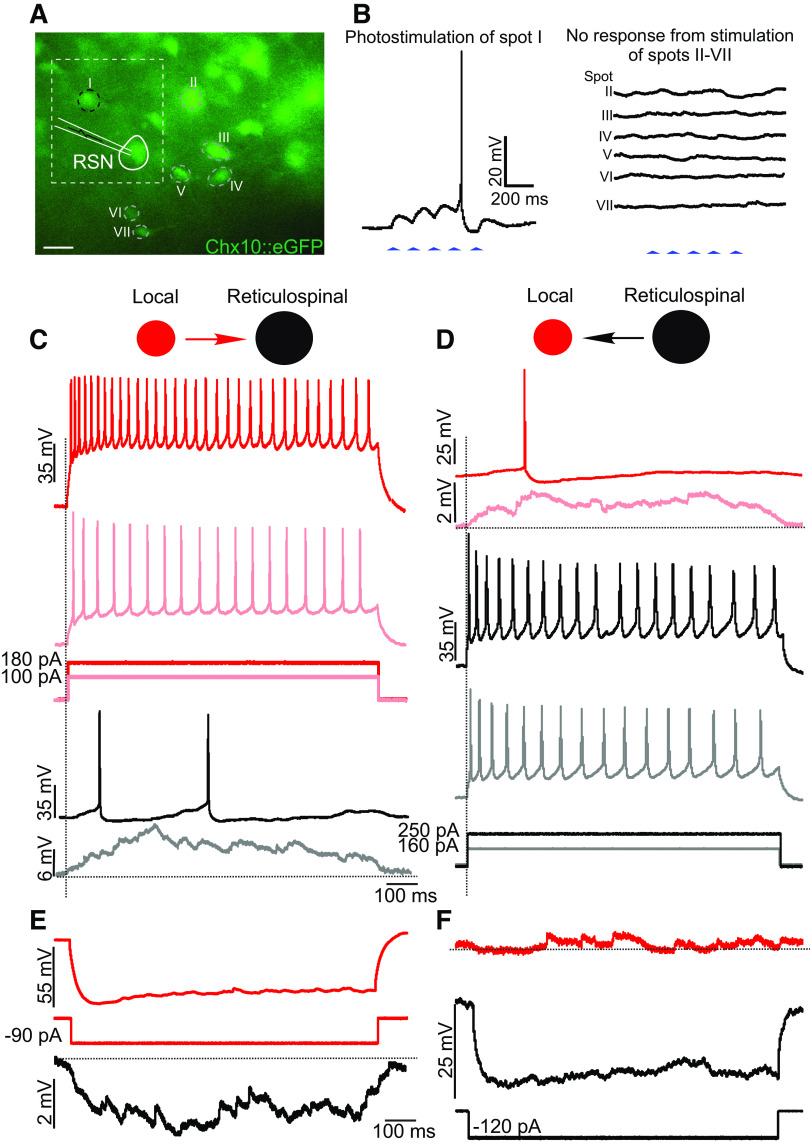
Bidirectional connectivity between a subset of Chx10 local interneurons and Chx10 reticulospinal neurons. *A*: fluorescence image of Chx10::eGFP positive neurons in the rostral GRN. Synaptic connectivity was determined by photolysis over visually identified Chx10 local interneurons while recording excitatory postsynaptic potentials (EPSPs) in the patched Chx10 RSN. Scale bar = 30 µm. *B*: photostimulation of *neuron 1* elicited EPSPs that summated to produce an action potential in the Chx10 reticulospinal neuron (RSN). After establishing this synaptic connection, a second electrode was used to patch and record responses in the Chx10 local interneuron (*neuron 1*). Note that electrophysiological properties of these two neurons are shown by the red dots in Fig. 1*E*. *C*: depolarizing current injections in the Chx10 local interneuron resulted in action potentials in the Chx10 local interneuron (red) and EPSPs and action potentials in the Chx10 RSN (black) at low- and high-current intensities, respectively. *D*: similarly, depolarizing current injections in the Chx10 RSN resulted in action potentials in the Chx10 RSN, and EPSPs and action potentials in the Chx10 local interneuron at low- and high-current intensities, respectively. *E*: hyperpolarizing current injections in the Chx10 local interneuron (red) resulted in a small hyperpolarizing response in the Chx10 RSN (black). *F*: hyperpolarizing current injections in the Chx10 RSN (black) did not elicit a detectable response in the Chx10 local interneuron (red). Note that the RSN could only be stably hyperpolarized by ∼25 mV. Horizontal dotted lines show baselines, and vertical dotted lines depict onsets of current injections. Example from *n* = 3 dual patch recordings from three mice of both sexes.

In two paired recording experiments, however, bidirectional connectivity was found using current injections into the identified presynaptic Chx10 local interneuron and Chx10 RSNs (Fig. 5, *A* and *B*). Incremental depolarizing current steps injected in Chx10 local interneurons (Fig. 5*C*) that generated action potentials led to EPSPs and action potentials in Chx10 RSNs (light gray and black, Fig. 5*C*) at low (light red, Fig. 5*C*) and high (dark red, Fig. 5*C*) current injections, respectively. Similarly, current injection in Chx10 RSNs (Fig. 5*D*) that generated action potentials also generated EPSPs and an action potential (light and dark red, Fig. 5*D*) in the Chx10 local interneuron at low (light gray, Fig. 5*D*) and high current injections (black, Fig. 5*D*), respectively, demonstrating that in at least some cases, connectivity is bidirectional.

Given this bidirectionality and given that Cx36 is widely expressed in the medRF GRN even into adulthood (34, 35), we sought to determine if these connections could have an electrical component, by delivering hyperpolarizing current injections. When Chx10 local interneurons were hyperpolarized, there was indeed a small hyperpolarizing response in Chx10 RSNs (*n* = 2, Fig. 5*E*), suggesting an electrical component to this connection. However, hyperpolarizing current steps injected in Chx10 RSNs failed to elicit a concomitant response in Chx10 local interneurons (Fig. 5*F*). The failure to evoke a hyperpolarizing response in the Chx10 local interneuron could be a result of a rectifying electrically coupled connection (36, 37) or could reflect that we were unable to hyperpolarize the Chx10 RSNs sufficiently to detect a response in the Chx10 local interneuron, as Chx10 RSNs became unstable during sustained hyperpolarization.

### Chx10 Local Interneurons Contribute to Repetitive Firing Behavior of Chx10 Reticulospinal Neurons

**Figure 6.**
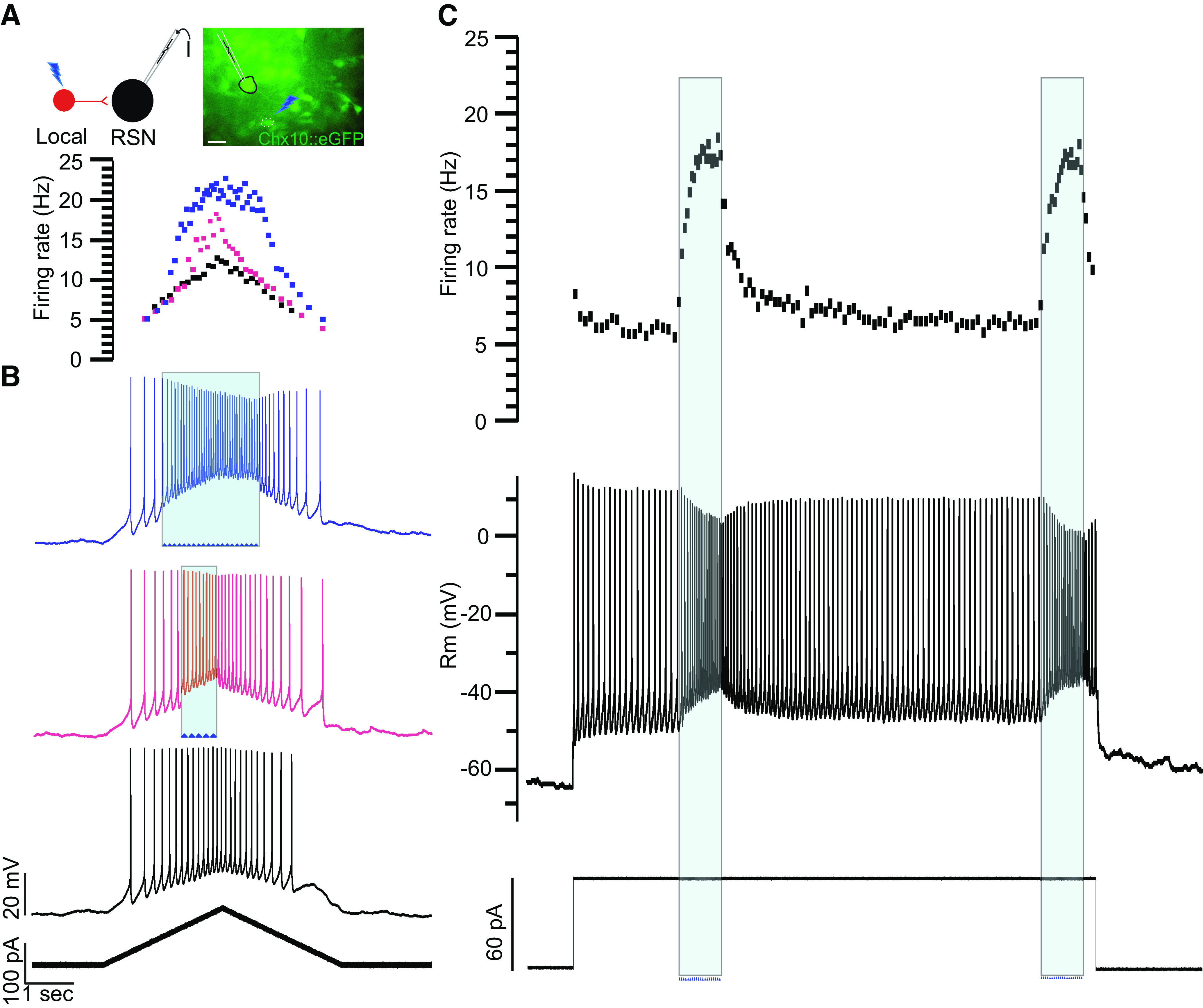
Chx10 local interneurons mediate the excitability of Chx10 reticulospinal neurons. *A*: a schematic of photolysis over a Chx10 local interneuron during recording and current injection in a Chx10 reticulospinal neuron (RSN). Scale bar = 30 µm. *B*: a Chx10 RSN (*inset* showing patched neuron) was subjected to ramp current injections (*bottom trace*) without (black trace) and with (red and blue traces) photostimulation of a connected Chx10 local interneuron (blue arrowheads and shading). In the *middle trace* (red), the photostimulation began later and lasted a shorter period of time than in the *top trace* (blue). The firing rate of the RSN was plotted with and without photostimulation (*top trace* in corresponding colors). *C*: same connected Chx10 local interneuron photostimulated (shaded blue) during sustained depolarizing current injection into the Chx10 RSN, with the resulting firing rate shown in the *top trace*. Example from *n* = 3 cells from three mice of both sexes.

To determine the degree to which inputs from Chx10 local interneurons impact the firing behavior of Chx10 RSNs, we photostimulated Chx10 local interneurons while inducing firing in Chx10 RSNs (*n* = 3, Fig. 6*A*). After a connection was established, Chx10 RSNs were subjected to ramp current injections alone and during photolysis over a connected Chx10 local interneuron (Fig. 6*B*). At its peak, photostimulation could double the firing rate of the Chx10 RSN, increasing peak firing rate from 10 ± 2 Hz to 22 ± 3 Hz. Similar increases in firing rate were seen when Chx10 local interneurons were photostimulated during rectangular depolarizing current (Fig. 6*C*). The average firing rate of 7 ± 2 Hz doubled to 15 ± 4 Hz during photostimulation of the connected Chx10 local interneuron. Interestingly, these induced firing rates are higher than those seen with maximal current injections into the recorded neurons (Fig. 1, *F*/*I* plots). The physiology underlying these photostimulation-induced firing patterns—acceleration during the stimulus and slow return to baseline—is unclear, but the patterns are similar to those seen with activation of persistent currents in motoneuron dendrites (38).

## DISCUSSION

The medullary reticular formation is an important site in the central nervous system for initiating and regulating movement (8, 21, 39, 40). This region—and in particular the GRN—has been studied primarily in terms of “command,” or reticulospinal, neurons. For example, in 1859, Mauthner cells were identified in fish, where they were found to initiate turning movement (41). Chx10 neurons in the *Xenopus* tadpole reticular formation are involved in initiating bouts of swimming (42). In cats and mice, excitatory neurons in this region receive inputs from higher centers involved in movement, including the MLR (8, 15) and the cerebellum (40). In recent years, neurons of the Chx10 lineage in the medRF have been identified as key instigators of movement (19, 22). But to understand how the medRF contributes to movement, it is important to understand whether this region serves mainly to integrate supramedullary motor commands for transmission to the spinal cord, or whether there is also local circuit processing. In this study, we demonstrated that there are at least two anatomically, physiologically, and functionally distinctive subtypes of Chx10 neurons in the rostral GRN, one with large somata that is reticulospinal, and the other with smaller somata that comprised of locally projecting excitatory interneurons. Using holographic photolysis of caged glutamate, we found that Chx10 local interneurons frequently formed local connections with other Chx10 local interneurons as well as with Chx10 RSNs, and that these local interneurons can facilitate the excitability of the RSNs. We propose that GRN Chx10 local interneurons are involved in processing and regulating the input:output functions of Chx10 RSNs.

### Subtypes of GRN Chx10 Neurons

It is not surprising that the GRN comprises heterogeneous neuronal subtypes. Electrophysiological analysis of neurons in the reticular formation of macaques has similarly demonstrated several (likely 4) different clusters of neurons based on their firing properties (43). Only one of these clusters was thought to be reticulospinal, suggesting the presence of other local or ascending neurons. Taken together with our data, it seems likely that distinct electrophysiological phenotypes in the reticular formation reflect different targets and functions of the recorded neurons.

Chx10, or Vsx2, is a transcriptional regulator that is expressed in some glutamatergic neurons in both the spinal cord and brain stem, as well as in other sites (e.g., retina). In the spinal cord, it is expressed in V2a neurons—the excitatory neurons of the cardinal V2 class. It is clear that spinal V2a neurons are not molecularly, physiologically, or functionally homogeneous: for example, lumbar V2a neurons have diverse electrophysiological properties (44) and only a subset of cervical V2a neurons projects to the brain stem (26). We had also previously demonstrated heterogeneity of Chx10^ON^ neurons in the reticular formation (15). But we now definitively demonstrate that there are at least two major subtypes of Chx10 neurons in the GRN—reticulospinal neurons and local interneurons.

Although we have shown that we can readily classify Chx10^ON^ GRN neurons into these two populations, it would not be surprising to find additional subclasses of these neurons. In the spinal cord, it is increasingly clear that cardinal neuronal classes are not homogeneous and that subtypes have different electrical properties, connectivity, and function [e.g., V0s (45–47), V1s (48), V2s (18, 49), V3s (32, 50, 51)]. In the reticular formation, we know that some GRN Chx10 neurons are involved in respiration (16, 52, 53), and others in locomotion. In the rostral GRN, bilateral excitation of Chx10 neurons leads to halting of locomotion through inhibition of locomotor circuits (19), and unilateral excitation leads to turning (22). With Chx10 RSNs, it has recently been shown that there are two subpopulations: those projecting to the cervical cord that lead to changes in head orientation and those that project to the lumbar cord that lead to changes in locomotor velocity (24). But we could not detect electrophysiological subtypes of Chx10 RSNs.

### Possible Roles of GRN Chx10 Local Interneurons in Sculpting Motor Commands

We have identified local excitatory interneurons in the GRN that form synaptic connections with RSNs in the region. This connections were seen at an early age (second postnatal week), at a time when mice are becoming increasingly mobile and pursuing goal-directed movements (30). Although we have not determined the role of these interneurons in behavior, it is interesting to consider how these local circuits might impact movement, as well as methodologies to investigate this question further.

Currently, there are no genetic determinants that can separate Chx10 local interneurons from Chx10 RSNs, and these neurons are anatomically comingled. Although optogenetic experiments have been used to sort out the role of Chx10 reticular formation neurons (19, 24, 26), such an approach would activate Chx10 interneurons and RSNs, obscuring the role of subpopulations. RSNs can be labeled or manipulated through retrograde viruses, for example see Ref. 24, but the function of interneurons would not be determined by doing so.

One way to parse function would be to record the activity of Chx10 neurons during behavior, noting that the smaller neurons are likely to be interneurons. Calcium imaging has recently been used to study the activity of Chx10 neurons in the GRN, finding that about one half are active during locomotor stop events. But there is diversity in function: that is, individual neurons are not active during every stop event, and some Chx10 neurons were active at the onset of and during locomotion (28). This finding is consistent with the in vitro demonstration of differential responses of Chx10 GRN neurons to stimulation of the mesencephalic locomotor region (15). And some of these neurons are not active during locomotion, but are during grooming, or when the animal is stationary (28). Similarly, three functional types of glutamatergic RSNs were described in the lamprey, in a similar region of the reticular formation (one that receives inputs from the mesencephalic locomotor region). Each of these neuronal types has a distinct function: initiation, maintenance, or stopping swimming (54, 55). That is, it is clear that reticular formation (GRN) neurons have diverse functions in behavior, but the degree to which this diversity reflects interneuron versus RSN activity is not clear.

In general, less attention has been paid specifically to local circuitry in the GRN. Local inhibitory circuits could facilitate bilateral coordination, as in Mauthner cells (56), or could conceivably aid in tuning descending commands. But the role of local excitatory interneurons is not obvious. Perhaps insight on the role of these interneurons could be gleaned through comparison with spinal circuits. In the spinal cord, several populations of excitatory interneurons are necessary for the production of normal coordinated activity, such as locomotion (see Ref. 57). If the GRN was simply for integration of descending inputs from various movement centers to produce descending commands, then local inhibitory interneurons may be sufficient to tune the commands. But if the GRN plays a role in the selection and/or generation of complex movement, then local circuitry—including excitatory interneurons—may be required. We show here that activating Chx10 interneurons leads to changes in RSN properties: firing rates in RSNs were higher than could be elicited by current injection, suggesting that dendritic inputs could drive these cells into a nonlinear range, as has been observed in other neurons such as spinal motoneurons (38). That is, interneuronal activity could change the nature of the descending commands—for example, sculpting tonic to phasic activity.

Several studies have modeled the internal organization of the reticular formation and its selection of appropriate RSNs for a motor behavior (58, 59). It has been suggested that local nodes of small interneurons (although in their case inhibitory) form synapses with reticulospinal neurons, and are organized through connections with other nearby interneurons within their node. This “small-world” network was hypothesized to ensure rapid cross-network synchronization, consistent node selection for a particular motor action, and increased persistent activity that facilitates ongoing drive required for the appropriate motor response (59). These properties were proposed to be consistent with a role of the GRN in action selection (60). Although these models used inhibitory interneurons, the concept of small-world networks may be generalizable to the architecture that we have demonstrated here. (The interested reader is directed to the clear analysis of Ref. 60, for further reading.)

In summary, we have shown that there are at least two distinct subtypes of Chx10 neurons in the rostral GRN: a local interneuronal and a reticulospinal population. And we have shown connectivity between these, such that the local interneurons enhance the firing of RSNs. We do not yet know how these circuits function to produce behavior, although we can propose that these local circuits are important for ensuring that the actions selected by higher motor circuits are processed to ensure that spinal circuits produce those behaviors.

## GRANTS

This work was funded by the Canadian Institutes of Health Research Grants MOP 136981 (to R. M. Brownstone), MOP-110950 (to Y. Zhang) and Wellcome Trust Grant 110193 (to R. M. Brownstone). R. M. Brownstone’s position is supported by Brain Research UK. Equipment used for photostimulation experiments was funded by the Canadian Foundation for Innovation Grant LOF 228190 (to R. M. Brownstone) and the Nova Scotia Research and Innovation Trust (to R. M. Brownstone).

## DISCLOSURES

R.M.B. is a cofounder of Sania Therapeutics, Inc., a gene therapy company.

## AUTHOR CONTRIBUTIONS

J.W.C. and R.M.B. conceived and designed research; J.W.C. performed experiments; J.W.C. analyzed data; J.W.C. and R.M.B. interpreted results of experiments; J.W.C. prepared figures; J.W.C. and R.M.B. drafted manuscript; J.W.C., Y.Z., and R.M.B. edited and revised manuscript; J.W.C., Y.Z., and R.M.B. approved final version of manuscript.
